# Histone deacetylase inhibitors induce apoptosis in human eosinophils and neutrophils

**DOI:** 10.1186/1476-9255-7-9

**Published:** 2010-02-04

**Authors:** Hannu Kankaanranta, Mirkka Janka-Junttila, Pinja Ilmarinen-Salo, Kazuhiro Ito, Ulla Jalonen, Misako Ito, Ian M Adcock, Eeva Moilanen, Xianzhi Zhang

**Affiliations:** 1 The Immunopharmacology Research Group, Medical School, FIN-33014, University of Tampere and Research Unit, Tampere University Hospital, Tampere, Finland; 2 Department of Respiratory Medicine, Seinäjoki Central Hospital, Seinäjoki, Finland; 3 Airway Disease, Imperial College School of Medicine at the National Heart and Lung Institute, London, UK

## Abstract

**Background:**

Granulocytes are important in the pathogenesis of several inflammatory diseases. Apoptosis is pivotal in the resolution of inflammation. Apoptosis in malignant cells is induced by histone deacetylase (HDAC) inhibitors, whereas HDAC inhibitors do not usually induce apoptosis in non-malignant cells. The aim of the present study was to explore the effects of HDAC inhibitors on apoptosis in human eosinophils and neutrophils.

**Methods:**

Apoptosis was assessed by relative DNA fragmentation assay, annexin-V binding, and morphologic analysis. HDAC activity in nuclear extracts was measured with a nonisotopic assay. HDAC expression was measured by real-time PCR.

**Results:**

A HDAC inhibitor Trichostatin A (TSA) induced apoptosis in the presence of survival-prolonging cytokines interleukin-5 and granulocyte-macrophage colony stimulating factor (GM-CSF) in eosinophils and neutrophils. TSA enhanced constitutive eosinophil and neutrophil apoptosis. Similar effects were seen with a structurally dissimilar HDAC inhibitor apicidin. TSA showed additive effect on the glucocorticoid-induced eosinophil apoptosis, but antagonized glucocorticoid-induced neutrophil survival. Eosinophils and neutrophils expressed all HDACs at the mRNA level except that HDAC5 and HDAC11 mRNA expression was very low in both cell types, HDAC8 mRNA was very low in neutrophils and HDAC9 mRNA low in eosinophils. TSA reduced eosinophil and neutrophil nuclear HDAC activities by ~50-60%, suggesting a non-histone target. However, TSA did not increase the acetylation of a non-histone target NF-κB p65. c-jun-N-terminal kinase and caspases 3 and 6 may be involved in the mechanism of TSA-induced apoptosis, whereas PI3-kinase and caspase 8 are not.

**Conclusions:**

HDAC inhibitors enhance apoptosis in human eosinophils and neutrophils in the absence and presence of survival-prolonging cytokines and glucocorticoids.

## Background

Eosinophils are important inflammatory cells involved in the pathogenesis of asthma and exacerbations of chronic obstructive pulmonary disease (COPD) [1]. Accumulation and activation of neutrophils at the inflamed site is involved in the pathogenesis of COPD, severe asthma and asthma exacerbations [1]. The process of apoptosis of granulocytes is believed to be pivotal in the resolution of inflammation, since it determines the rapid clearance of intact senescent eosinophils and neutrophils, thus providing an injury-limiting granulocyte clearance mechanism [2,3]. Eosinophil and neutrophil apoptosis can be modulated by glucocorticoids and death receptors i.e. Fas and inhibited by survival-prolonging cytokines such as interleukin-5 (IL-5) and granulocyte-macrophage colony-stimulating factor (GM-CSF) [2,3]. We, and others, have previously shown that eosinophil apoptosis is delayed in patients with asthma or inhalant allergy [4-6]. However, the mechanisms of apoptosis in these cells remain largely unknown. In fact, it is not even known whether the main event controlling eosinophil apoptosis is upregulation or downregulation of genes [3].

Histone acetylation regulates inflammatory gene expression and also plays a role in diverse functions such as DNA repair and cell proliferation and apoptosis [7,8]. In the resting cell, DNA is tightly compacted around core histones. Specific residues within the N-terminal tails of histones can be posttranslationally modified by acetylation, leading to release of the tightly wound DNA. Conversely, histone deacetylation is thought to re-establish the tight nucleosomal structure [7,8]. Histone acetylation is regulated by a dynamic balance between histone acetyltransferases (HAT) and histone deacetylases (HDAC). Changes in histone acetylation patterns have been reported in many human diseases, particularly cancer, and investigators have used HDAC inhibitors against many malignancies. HDAC inhibitors induce apoptotic cell death in a number of tumor cell types [9,10]. In contrast, normal cells are usually resistant to cell death caused by HDAC inhibitors [9,10].

However, recent in vivo data in animal models suggest that HDAC inhibitors may have potential to act as anti-inflammatory and anti-allergic agents. For example, evidence from an adjuvant-induced arthritis-model suggests that HDAC inhibitors may be useful in rheumatoid arthritis [11]. Recently, Choi and coworkers [12] demonstrated that trichostatin A (TSA) blocked ovalbumin (OVA) -induced airway hyper-responsiveness, as well as reduced the numbers of eosinophils in lavage fluid. Even though HDAC inhibitors do not usually induce apoptosis in non-malignant cells, the promising in vivo findings prompted us to test the effects of HDAC inhibitors on apoptosis of terminally differentiated primary cells such as human eosinophils and neutrophils.

## Methods

### Blood donors

For neutrophil experiments blood was obtained from healthy donors. For eosinophil experiments, blood (50-100 ml) was obtained from eosinophilic individuals. However, patients with hypereosinophilic syndrome were excluded. All subjects gave informed consent to a study protocol approved by the ethical committee of Tampere University Hospital (Tampere, Finland).

### Neutrophil and eosinophil isolation

Neutrophils from venous blood were isolated under sterile conditions as previously reported [13,14]. Neutrophil populations with purity of >98% were accepted for the experiments. The neutrophils were resuspended at 2 × 10^6 ^cells/ml, cultured for 16 h (37°C; 5% CO_2_) in RPMI 1640 (Dutch modification) with 10% fetal calf serum plus antibiotics. Eosinophils were purified by using immunomagnetic anti-CD16 antibody conjugated beads as previously described [5,15-17]. The purity of eosinophil population was > 99%. The eosinophils were resuspended at 1 × 10^6 ^cells/ml, cultured (37°C, 5% CO_2_) for 18 h (morphological and Annexin-V assays) or 40 h (relative DNA fragmentation assay) in the absence or presence of cytokines, glucocorticoids and HDAC inhibitors in RPMI 1640 (Dutch modification) with 10% fetal calf serum plus antibiotics in 96-well plates.

### Macrophage cultures

J774.2 macrophages (The European Collection of Cell Cultures, Porton Down, Wiltshire, UK) were cultured at 37°C, 5% CO2 atmosphere, in Dulbecco's Modified Eagle's Medium with Ultraglutamine 1 (DMEM/U1) supplemented with 5% of heat inactivated foetal bovine serum, penicillin (100 U/ml), streptomycin (100 μg/ml) and amphotericin B (250 ng/ml). Cells were seeded on 24 well plates and grown to confluence prior to experiments. Cells were cultured for 24 h in the presence or absence of various concentrations of TSA or lipopolysaccharide (LPS; 10 ng/ml) and ammonium pyrrolidinedithiocarbamate (PDTC; 100 μM), whereafter medium was removed, cells were washed once with phosphate-buffered saline (PBS) and double-stained with Annexin-V and PI.

### Apoptosis assays

Apoptosis was determined by propidium iodide staining of DNA fragmentation and flow cytometry (FACScan, Becton Dickinson, San Jose, CA) as previously described [15-17]. The cells showing decreased relative DNA content were considered apoptotic [15,16]. Annexin V-binding assay was performed as previously described [14,16] and cells showing positive staining with Annexin-V (i.e. both early apoptotic Annexin V^+ve^/PI^-ve ^and late apoptotic/secondary necrotic cells: Annexin V^+ve^/PI^+ve^) were considered to be apoptotic. For morphological analysis, eosinophils or neutrophils were centrifuged onto cytospin slides (1000 rpm, 7 min) and stained with May-Grünwald-Giemsa after fixation in methanol. The cells showing typical features of apoptosis such as cell shrinkage, nuclear coalescence and nuclear chromatin condensation were considered as apoptotic [5,15,16].

### Western blotting

Eosinophils were suspended at 10^6 ^cells/ml and cultured at +37°C for 1 h in the absence and presence of DMSO (solvent control), TSA (330 nM) or GM-CSF (0.1 ng/ml). Thereafter the samples were centrifuged at 1000 g for 1 min. The cell pellet was lysed by incubating for 15-30 min in 40 μl of ice-cold RIPA buffer with protease inhibitors. The sample was centrifuged at 12000 g for 5 min and the debris was carefully removed. Samples were mixed into SDS (sodium dodecyl sulfate)-containing loading buffer and stored at -20°C until the Western blot analysis. The protein sample (25-30 μg) was loaded onto 10% SDS-polyacrylamide electrophoresis gel and electrophoresed for 2 h at 120 V. The separated proteins were transferred to Hybond enhanced chemiluminescence nitrocellulose membrane (Amersham Biosciences UK, Ltd., Little Chalfont, Buckinghamshire, UK) with a semidry blotter at 2 mA cm^-2 ^for 60 min. After transfer, the membranes were blocked by 5% bovine serum albumin (BSA) in TBST (20 mM Tris base pH 7.6, 150 mM NaCl, 0.1% Tween-20) for 1 h at room temperature and incubated with the specific primary antibody overnight at +4°C in the blocking solution. The membrane was thereafter washed 3× with TBST for 5 min, incubated for 30 min at room temperature with the secondary antibody in the blocking solution and washed 3× with TBST for 5 min. Bound antibody was detected by using SuperSignal West Dura chemiluminescent substrate (Pierce, Cheshire, UK) and FluorChem 8800 imaging system (Alpha Innotech Corporation, San Leandro, CA, USA). The chemiluminescent signal was quantified by using the FluorChem software version 3.1.

### HDAC colorimetric activity assay

Nuclear extracts were prepared from 5 × 10^6 ^cells using a modification of method of Dignam et al [18]. Briefly, isolated cells were washed with cold PBS and suspended in hypotonic buffer A (20 mM HEPES-KOH, pH 7.9, 3.0 mM MgCl_2_, 20 mM KCl and protease inhibitor mixture). After incubation for 30 min on ice, 0.2 volumes of 10% igepal CA-30 (v/v) was added, and the cells were vortexed for 30 s. Eosinophils were further processed by Dounce tissue homogenizer. Following centrifugation at 12,000 g for 10 s, the supernatant was discarded and the pellet was washed in 100 μl of buffer A without Igepal and re-centrifuged. The pelleted nuclei were resuspended in buffer C (40 mM HEPES-KOH, pH 7.9, 50% glycerol, 840 mM NaCl, 3 mM MgCl_2_, 0.2 mM EDTA and protease inhibitor cocktail tablet solution) and incubated for 20 min on ice. Nuclei were vortexed for 1 min and nuclear extracts were obtained by centrifugation at 12,000 g for 2 min, 4°C and stored at -76°C until use.

HDAC colorimetric activity assay was carried out according to the manufacturer's instructions. HDAC inhibitors and assay buffer were mixed to the wells of the microtiter plate. Nuclear extracts were added to appropriate wells and equilibrated to assay temperature (37°C). Color de Lys™ substrate was added and mixed in each well to initiate HDAC reactions and incubated at 37°C for 30 min. Color de Lys™ developer was added to stop HDAC reaction. The mixture was incubated at 37°C for 15 min and read in microtiter-plate reader (Wallac, Turku, Finland) at 405 nm.

### Real-time PCR

To isolate mRNA from human eosinophils and neutrophils, the cells were first sedimented whereafter TRI REAGENT (1.0 ml/5 × 10^6 ^eosinophils) was added. mRNA was isolated according to the manufacturer's instructions and reverse transcription of RNA to cDNA was performed as described previously [19].

Gene transcript levels of HDAC1 to 11 and the housekeeping genes glyceraldehydes-3 phosphate dehydrogenase (GAPDH) and GLB2L1 were quantified by real-time PCR using a Taqman master mix (Applied Biosystems, Foster City, CA) on a Rotor-Gene 3000 PCR apparatus (Corbett Research, N.S.W., Australia). The primer pairs were purchased from Applied Biosystems. Variations in cDNA concentration between different samples were corrected using the housekeeping gene. The relative amount of gene transcript present was calculated and normalized by dividing the calculated value for the gene of interest by the housekeeping gene value.

### Materials

Reagents were obtained as follows: apicidin, MC-1293 and MS-275 (Alexis, Lausen, Switzerland), CD95 monoclonal antibody (clone CH-11; Immunotech, Marseille, France), NF-kB p65 and acetyl-NF-kB p65 (Lys310) antibodies (Cell Signaling Technology, Inc., Danvers, USA), fluticasone, igepal CA-630, LPS, PDTC and trichostatin A (Sigma Chemical Co., St. Louis, MO, USA), Z-VE(OMe)ID(OMe)-FMK, Z-D(OMe)QMD(OMe)-FMK, IETD-CHO, Q-VD-OPh and LY294002 (Calbiochem, San Diego, USA), HDAC colorimetric activity kit (Biomol, Plymouth Meeting, USA), mometasone (Schering-Plough, Kenilworth, NJ), DMEM/U1 (Lonza Verviers SPRL, Verviers, Belgium), penicillin, streptomycin and amphotericin (Invitrogen, Paisley UK), wortmannin (Merck, Darmstadt, Germany) and TRI REAGENT (Molecular Research Center, Inc., Cincinnati, OH). Other reagents were obtained as previously described [5,13-17,19]. Stock solutions of budesonide (50 mM) were prepared in ethanol. The final concentration of ethanol in the culture was 0.2%. Stock solutions of HDAC inhibitors were prepared in DMSO. The final concentration of DMSO in the culture was 0.5%. A similar concentration of DMSO was used in control experiments.

### Statistics

Results are expressed as Mean ± SEM. The EC_50 _was defined as the concentration of drug producing 50% of its maximal effect. Statistical significance was calculated by analysis of variance for repeated measures supported by Student-Newman-Keuls multiple comparisons test or Dunnett test. HDAC expression levels obtained by quantitative PCR were compared using Mann-Whitney U-test. Differences were considered significant when P < 0.05.

## Results

### HDAC inhibitors enhance eosinophil apoptosis in the presence of survival-prolonging cytokines

**Figure 1:**
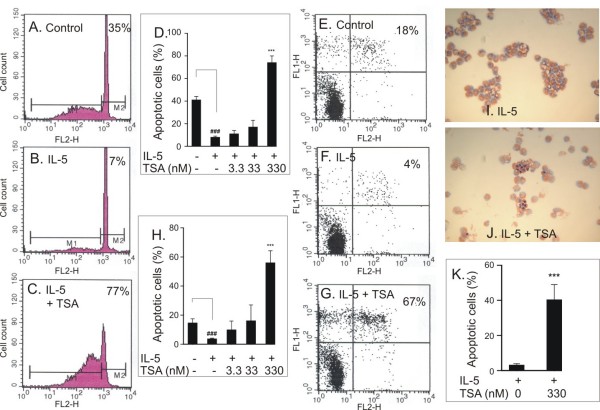
**The effect of TSA (330 nM in C, G, J, K) on eosinophil apoptosis in the presence of IL-5 (0.3 ng/ml) as measured by relative DNA fragmentation assay (A-D), Annexin V binding assay (E-H; Annexin V-FITC: FL1-H and propidium iodide: FL2-H)) and morphological analysis (I-K)**. Figures in top right hand corner represent the percentage of eosinophils showing decreased relative DNA content (A-C) or total percentage of apoptotic eosinophils (all Annexin V-FITC^+ve ^cells) (E-G). In A-C, E-G and I-J a representative of 6 similar experiments is shown. Mean ± SEM, n = 6 (D, H, K). ***P < 0.001 vs. solvent control in the presence of IL-5 and ### P < 0.001 vs. the control in the absence of IL-5 and TSA.

IL-5 inhibited human eosinophil apoptosis in a concentration-dependent manner and maximal inhibition of apoptosis was obtained at 0.3 ng/ml concentration (percentage of apoptotic cells 41 ± 3 and 8 ± 1 in the absence and presence of IL-5, respectively, n = 5, P < 0.001). TSA (330 nM) enhanced apoptosis in the presence of IL-5 as evidenced by an increase in the number of cells showing decreased relative DNA content (Figure 1A-C). The effect of TSA was concentration-dependent and the EC_50 _value for the enhancement of apoptosis in the presence of IL-5 was 92 ± 8 nM, n = 6; Figure 1D). This increase in the number of apoptotic cells was confirmed by showing increased phosphatidylserine expression on the outer leaflet of cell membrane of IL-5-treated cells, i.e. the percentage of Annexin-V-positive cells (Figure 1E-H). Furthermore, an increase in the number of eosinophils showing the typical morphological features of apoptosis such as nuclear coalescense, chromatin condensation and cell shrinkage was found with TSA (Figure 1-K).

**Figure 2:**
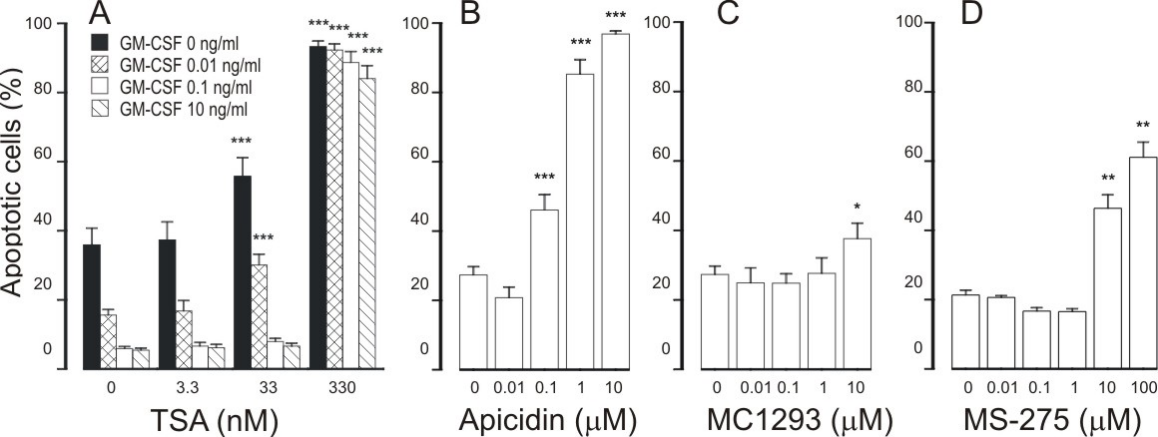
**The effect of HDAC inhibitors Trichostatin A (TSA; A), apicidin (B), MC1293 (C) and MS-275 (D) on eosinophil apoptosis in the presence of GM-CSF (in B-D: 0.1 ng/ml)**. In (A) the black colums indicate the effect of TSA in the absence of GM-CSF. Apoptosis was assessed by flow cytometry measuring the relative DNA fragmentation. *P < 0.05, **P < 0.01 and ***P < 0.001 as compared with the respective control. Mean ± S.E.M., n = 5-6.

To evaluate whether the effect of TSA is specifically related to IL-5, we employed another eosinophil survival-prolonging cytokine, i.e. GM-CSF. GM-CSF (0.01 - 10 ng/ml) promoted eosinophil survival in a concentration-dependent manner (Figure 2A). TSA (3.3-330 nM) enhanced apoptosis in the presence of GM-CSF (0.01 - 10 ng/ml) (Figure 2A, Table 1).

**Table 1:** The EC_50 _Values for the effects of trichostatin A on apoptosis in eosinophils and neutrophils.

	EC_50 _(nM)		
	
Apoptosis	Eosinophils	neutrophils	P value
GM-CSF			
0.01 ng/ml	79 ± 2		
0.1 ng/ml	102 ± 1		
10 ng/ml	93 ± 1	123 ± 9	0.0042
IL-5	92 ± 8		
Constitutive	34 ± 10	97 ± 22	0.0007
Budesonide	32 ± 17	99 ± 7	0.026
Fluticasone	47 ± 15	100 ± 11	0.017
Mometasone	20 ± 5	87 ± 9	< 0.0001
			
Fas	31 ± 10		

Values are the mean ± S.E.M. of six duplicate experiments with cells isolated from different donors.

Glucocorticoids are known to partially antagonize the survival-prolonging action of IL-5 or GM-CSF on eosinophils. However, this effect of glucocorticoids is abolished when the cytokine is used at higher concentrations [14,20-22]. For example, recently, we reported that budesonide (1 μM) partly antagonizes cytokine-afforded survival in the presence of low but not in the presence of high concentrations of IL-5 [16]. The maximal response and the EC_50 _values (Table 1) of TSA were almost similar independently of the concentration of GM-CSF, suggesting that the cellular targets of TSA are different from that of glucocorticoids.

To evaluate whether the ability to antagonize cytokine-afforded eosinophil survival is not related to TSA only, we employed other pharmacological inhibitors of HDACs. Another general HDAC inhibitor, apicidin (0.1 - 10 μM) antagonized GM-CSF-mediated eosinophil survival by inducing apoptosis with an EC_50 _of 427 ± 42 nM (Figure 2B). MC-1293, a commercially available HDAC1 inhibitor, antagonized GM-CSF-mediated eosinophil survival only partially at high (10 μM) drug concentrations (Figure 2C). Another HDAC inhibitor, MS-275 (0.1-1 μM), at concentrations known to inhibit HDAC1 [23] did not affect GM-CSF-afforded eosinophil survival. In contrast, at higher concentrations (10-100 μM) known to inhibit HDAC3 [23], MS-275 enhanced apoptosis in GM-CSF-treated eosinophils (Figure 2D).

### HDAC inhibitors enhance constitutive eosinophil apoptosis

In the absence of life-supporting cytokines, TSA increased the number of cells showing decreased relative DNA content suggesting apoptosis (Figure 2A, Table 1). Similarly, an increase in the number of cells presenting with the typical morphological features of apoptosis was found with TSA (percentage of apoptotic cells 11 ± 3 and 62 ± 8 in the absence and presence of 330 nM TSA, respectively, n = 5, P < 0.001). This was confirmed by showing an increase in the percentage of Annexin-V-positive cells in the absence and presence of TSA (330 nM) (15 ± 3% and 68 ± 8%, respectively, n = 6, P < 0.001).

**Figure 3:**
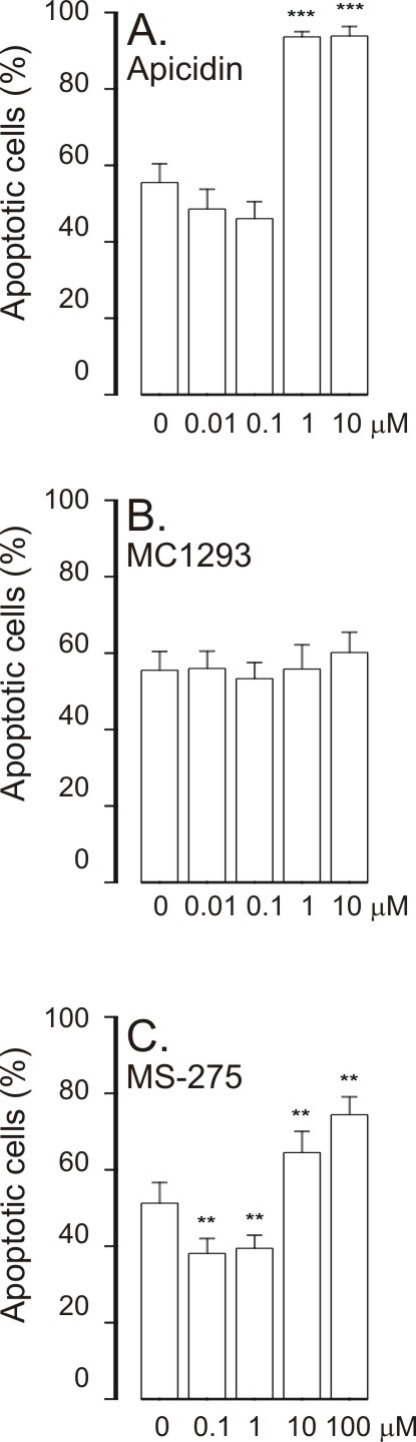
**The effect of HDAC inhibitors apicidin (A), MC1293 (B) and MS-275 (C) on apoptosis in eosinophils in the absence of survival-prolonging cytokines (ie. spontaneous apoptosis)**. Apoptosis was assessed by flow cytometry measuring the relative DNA fragmentation in propidium iodide-stained cells. **P < 0.01 and ***P < 0.001 as compared with the respective control in the absence of HDAC inhibitors. Mean ± S.E.M. of 5-6 independent determinations using cells from different donors.

Apicidin enhanced spontaneous eosinophil apoptosis (Figure 3A). The selective HDAC1 inhibitor, MC1293, did not enhance eosinophil apoptosis (Figure 3B). MS-275 (0.1-1 μM) inhibited constitutive eosinophil apoptosis slightly, but at higher concentrations (10-100 μM), known to inhibit HDAC3 [23], MS-275 enhanced constitutive eosinophil apoptosis (Figure 3C).

### HDAC inhibitors have additive effect on glucocorticoid-induced eosinophil apoptosis

**Figure 4:**
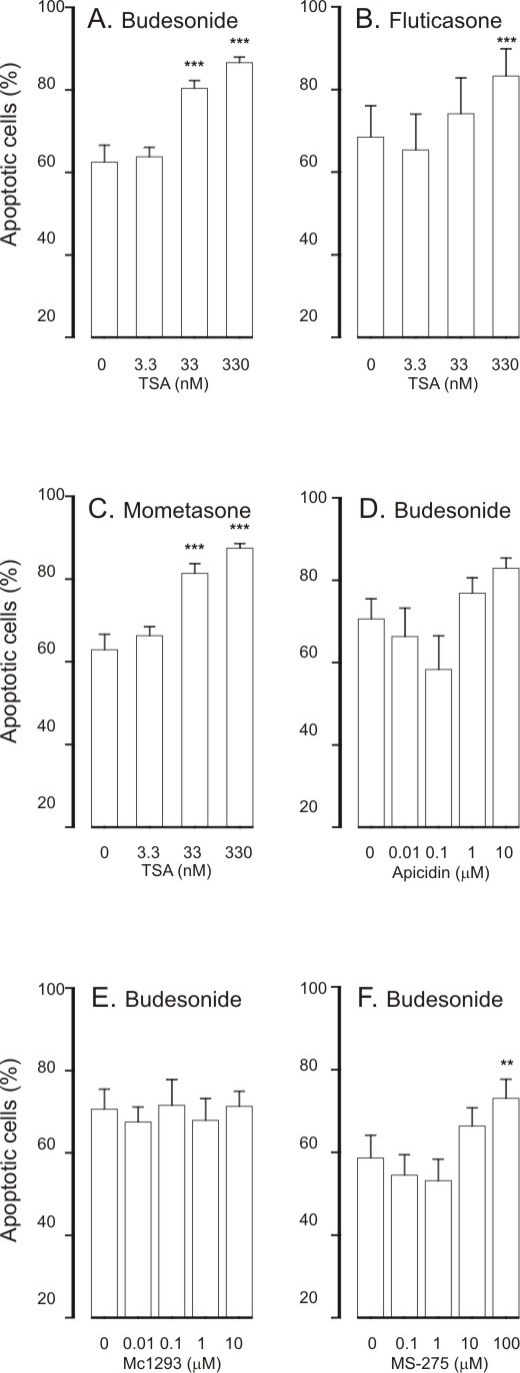
**The effect of trichostatin A (A-C) on human eosinophil apoptosis in the presence of budesonide (1 μM; A), fluticasone (1 μM; B) or mometasone (1 μM; C)**. In (D-F) is shown the effects of HDAC inhibitors apicidin (D), MC1293 (E) and MS-275 (F) on eosinophil apoptosis in the presence of budesonide (1 μM). Apoptosis was assessed by flow cytometry measuring the relative DNA fragmentation in propidium iodide-stained cells. ** indicates P < 0.01 and *** P < 0.001 as compared with the respective control in the absence of HDAC inhibitors. Mean ± S.E.M. of 5-6 independent determinations using cells from different donors. The corresponding percentage of apoptotic cells in the absence of glucocorticoids and HDAC-inhibitors was 49 ± 3 (n = 25).

Glucocorticoids increase apoptosis of human eosinophils at clinically relevant drug concentrations [3,14,20]. Budesonide, fluticasone and mometasone (all at 1 μM) enhanced constitutive eosinophil apoptosis (Figure 4A-C and figure legend). A general HDAC inhibitor, TSA (3.3-330 nM), had an additive effect in the presence of glucocorticoids (Figure 4A-C) on eosinophil apoptosis. The EC_50 _values of TSA for the enhancement of eosinophil apoptosis in the presence of glucocorticoids ranged from 20 ± 5 nM to 47 ± 15 nM (Table 1). The additive effect of TSA (3.3-330 nM) on budesonide-induced eosinophil apoptosis was confirmed by using morphological analysis and Annexin-V binding assay (n = 5-6, P < 0.05; data not shown).

Apicidin (1 nM-10 μM) also had an additive effect on budesonide-induced eosinophil apoptosis (Figure 4D). In contrast, MC-1293 (1 nM-10 μM, Figure 4E) failed to enhance budesonide-enhanced eosinophil apoptosis. MS-275 at higher concentrations (10-100 μM) had an additive effect on budesonide-induced eosinophil apoptosis (Figure 4F).

### HDAC-inhibitors have an additive effect on Fas-induced eosinophil apoptosis

Activation of Fas enhanced constitutive apoptosis of eosinophils (percentage of apoptotic cells 47 ± 4 and 65 ± 2 in the absence and presence of 100 ng/ml activating CD95 monoclonal antibody, respectively, n = 6, P < 0.01). TSA (3.3-330 nM) had an additive effect on Fas-induced eosinophils apoptosis (Table 1 and Table 2). This was confirmed by measuring the percentage of Annexin-V-positive cells in the absence and presence of TSA (330 nM) (36 ± 6% vs 74 ± 8%, n = 6, P < 0.001). Furthermore, an increase in the number of eosinophils showing the typical morphological features of apoptosis was found with TSA (percentage of apoptotic cells 26 ± 7 and 78 ± 7 in the absence and presence of 330 nM TSA, respectively, n = 6, P < 0.001).

**Table 2:** The effects of trichostatin A on Fas-induced eosinophil apoptosis.

	**Percentage of apoptotic cells**
	
Control	47 ± 4
Fas	65 ± 2##
Fas +trichostatin A 3.3 nM	67 ± 3
Fas +trichostatin A 33 nM	79 ± 2***
Fas +trichostatin A 330 nM	89 ± 1***

Shown is the percentage of apoptotic cells after 40 h incubation as analyzed by relative DNA fragmentation assay

Values are the mean ± SEM of independent determinations using cells from different donors. n = 6. *** indicates P < 0.001 as compared with the respective solvent control in the absence of trichostatin A and ## P < 0.01 as compared with the respective control in the absence of Fas. The concentration of Fas activating antibody was 100 ng/ml.

### Effect of HDAC inhibitors on neutrophil apoptosis

**Figure 5:**
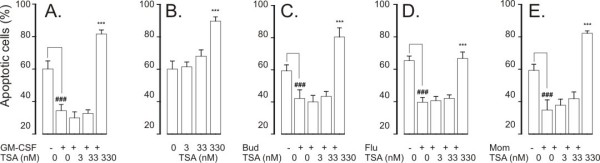
**The effect of Trichostatin A on apoptosis in human neutrophils in the presence (A) or absence (B) of the survival-prolonging cytokine GM-CSF (10 ng/ml)**. In (C-E) is shown the effect of trichostatin A on human neutrophil apoptosis in the presence of budesonide (1 μM; C), fluticasone (1 μM; D) or mometasone (1 μM; E). Apoptosis was assessed by flow cytometry measuring the relative DNA fragmentation assay. *** P < 0.001 as compared with the respective control in the absence of HDAC inhibitors. ### P < 0.001 as compared with the respective control in the absence of HDAC inhibitors and GM-CSF or glucocorticoids. Mean ± S.E.M. of 6 independent determinations using cells from different donors.

Neutrophils rapidly undergo apoptosis when cultured in the absence of survival-prolonging factors. GM-CSF inhibited constitutive apoptosis in neutrophils (percentage of apoptotic cells 60 ± 5 and 34 ± 4 in the absence and presence of 10 ng/ml GM-CSF, respectively, n = 6, P < 0.001). TSA (3.3-330 nM) antagonized the the survival promoting action of GM-CSF (Figure 5A) with an EC_50 _of 123 ± 9 nM. The enhancement of neutrophil apoptosis by TSA in the presence of GM-CSF was confirmed by annexin-V binding analysis (47 ± 5% vs 60 ± 8%, n = 4, P < 0.05). TSA also enhanced spontaneous neutrophil apoptosis 1.5-fold (Figure 5B).

In contrast to the enhancing effect on eosinphil apoptosis, glucocorticoids inhibit apoptosis in human neutrophils [13,14,24]. For example, budesonide inhibited neutrophil apoptosis, the percentages of apoptotic cells were 60 ± 5 and 42 ± 5 in the absence and presence of budesonide (1 μM), respectively (n = 6, P < 0.001, Figure 5C). TSA (3.3-330 nM) antagonized the inhibitory effect of budesonide (Figure 5C) on neutrophil apoptosis. This was confirmed by Annexin-V binding analysis (55 ± 4% vs 91 ± 1% Annexin V-positive cells, n = 6, P < 0.001). Furthermore, TSA antagonized fluticasone- (Figure 5D) and mometasone- (Figure 5E)-induced survival of neutrophils by inducing apoptosis. The EC_50 _values of TSA for antagonizing glucocorticoid-afforded survival in neutrophils were not different between the glucocorticoids (Table 1).

### Pharmacological nature of the effect of HDAC inhibitors

**Figure 6:**
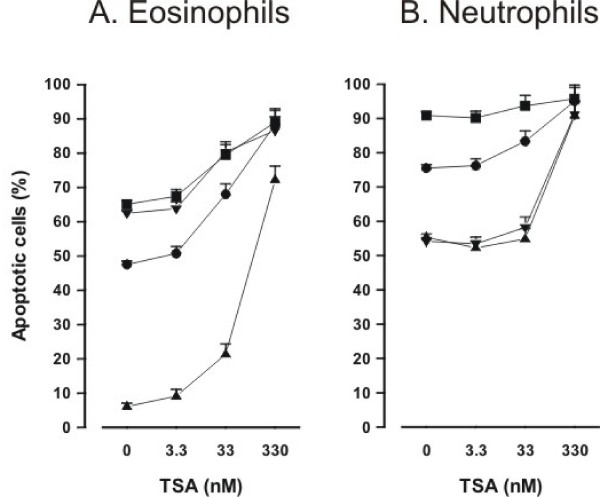
**Concentration-response curves of TSA in eosinophils (A) and neutrophils (B) in the absence (black circle) and presence of survival-prolonging cytokines (black up-pointing triangle; IL-5 0.3 ng/ml in eosinophils and GM-CSF 10 ng/ml in neutrophils), budesonide (black down-pointing triangle; 1 μM) or Fas (black square; 100 ng/ml)**. Apoptosis was assessed by flow cytometry measuring the relative DNA fragmentation (A) or Annexin V-binding (B). Eosinophils or neutrophils were isolated and concentration-response curves in the absence or presence of cytokines, budesonide or Fas were prepared simultaneously from the cells of the same donor. Mean ± S.E.M. of 6 independent determinations using cells from different donors.

To further evaluate whether the effects of HDAC inhibitors on eosinophil and neutrophil apoptosis in the presence of glucocorticoids or Fas are additive or synergistic, dose-response curves of TSA in the absence or presence of survival-prolonging cytokines, glucocorticoids and Fas are compared (Figure 6A and 6B). In eosinophils, the maximal percentage of apoptotic cells is similar in the presence of TSA (330 nM) alone and in the presence of budesonide and TSA (330 nM) (Figure 6A). This indicates that the effect is additive, but not synergistic. The same can be seen with the combination of TSA and Fas. Similarly, in neutrophils, the maximal percentage of apoptotic cells is similar in the presence of TSA (330 nM) alone and in the presence of Fas and TSA (330 nM) (Figure 6B). In neutrophils, TSA enhanced apoptosis in the presence of GM-CSF and budesonide in a similar manner within the same concentration range (Figure 6B). Similarly, in eosinophils TSA enhanced apoptosis in the presence of IL-5 (Figure 6A). This suggests that the antagonism of the actions of survival-prolonging cytokines IL-5 and GM-CSF in both cell types and the antagonism of the actions of glucocorticoids does not occur at the level of IL-5, GM-CSF or glucocorticoid receptors.

### HDAC expression in human eosinophils and neutrophils

**Figure 7:**
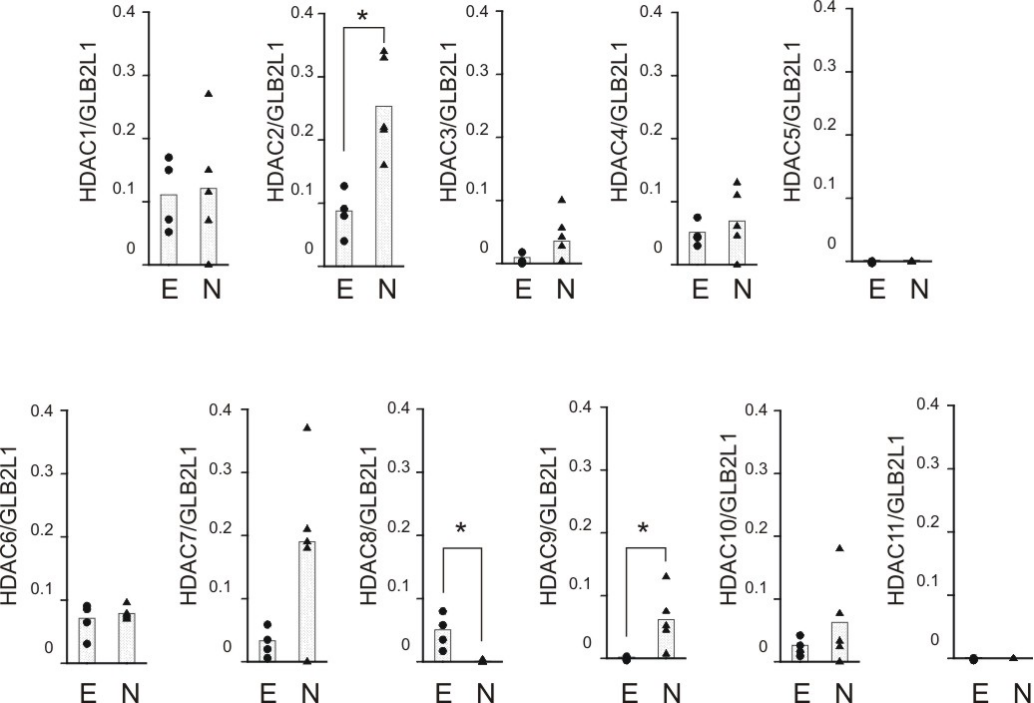
**The expression of histone deacetylases (HDAC) 1-11 in human eosinophils (black circle; E) and neutrophils (black up-pointing triangle; N)**. HDAC mRNA levels were normalized against GLB2L1 mRNA. Total mRNA from eosinophils (n = 4) and neutrophils (n = 5) was extracted and subjected to RT-PCR. *P < 0.05 for the difference between eosinophils and neutrophils.

To evaluate whether granulocytes express HDACs, we isolated mRNA from human eosinophils and neutrophils and measured the expression of different HDACs using real-time PCR. To confirm the accuracy of the results, the expression of different HDACs was normalized against two different housekeeping genes, namely GAPDH and GLB2L1. This analysis gave almost identical results. Expression of HDAC5, 9 and 11 was very low in eosinophils and expression of HDAC5, 8 and 11 was very low in neutrophils (Figure 7). The expression of HDAC2 and HDAC9 was higher in neutrophils than in eosinophils and the expression of HDAC8 was significantly higher in eosinophils (Figure 7).

### HDAC activity in eosinophils and neutrophils

**Figure 8:**
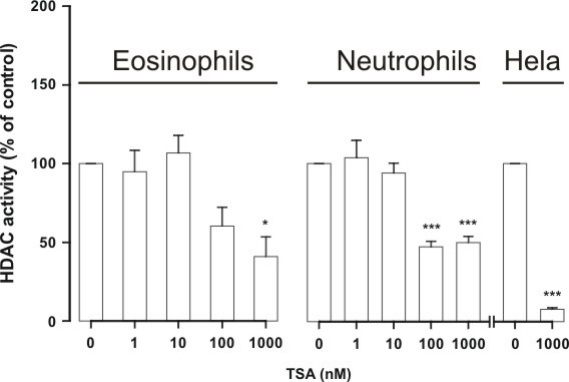
**The effect of HDAC inhibitor Trichostatin A (TSA) on HDAC activity in nuclear extracts isolated from human eosinophils (n = 6) and neutrophils (n = 5)**. For comparison is shown the effect of TSA on HDAC activity in HeLa nuclear extracts (n = 6). Nuclear extracts were prepared and HDAC activity was measured as described in materials and methods. HDAC activity in the absence of TSA was set as 100%. *P < 0.05 and *** P < 0.001 as compared with the respective control in the absence of TSA. Mean ± S.E.M.

The HDAC activity in eosinophil nuclear extracts was somewhat higher (0.37 ± 0.05 OD/mg/min; n = 6) than in neutrophil nuclear extracts (0.22 ± 0.05 OD/mg/min; n = 5, P < 0.05). For comparison, we included HeLa-cell nuclear extracts which had clearly higher HDAC activity (0.70 ± 0.04 OD/mg/min, n = 6, P < 0.001 versus eosinophil and neutrophil nuclear extracts). TSA inhibited substrate (1.25 mM) deacetylation by eosinophil and neutrophil nuclear extracts only partially. The maximal inhibition of HDAC activity by TSA (1000 nM) in eosinophil nuclear extracts was 59 ± 13% (n = 6, P < 0.05) and in neutrophil nuclear extracts it was 50 ± 4% (n = 5, P < 0.001), whereas in HeLa nuclear extracts HDAC activity was inhibited almost completely (93 ± 1% inhibition, n = 6, P < 0.001) by 1000 nM TSA (Figure 8).

### Acetylation of NF-κB p65 does not explain the apoptosis-inducing effect of TSA in human eosinophils

**Figure 9:**
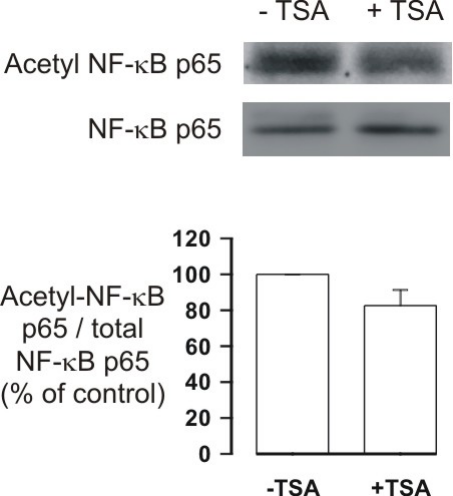
**The effect of TSA (330 nM) on the expression of acetyl-NF-kB p65 (Lys310)**. Human eosinophils were treated with solvent or TSA for 1 h and immunoblots were run using antibodies against acetyl-NF-kB p65 and total NF-kB p65. The chemiluminescent signal was quantified as described under Materials and Methods. Acetyl-NF-kB p65 values were normalized to NF-kB p65 values and the value in the absence of TSA was set as 100%. Results are expressed as mean ± S.E.M., n = 5.

The above data suggest that the effects of HDAC inhibitors in eosinophils or neutrophils may not be mediated via regulation of acetylation status of histones, but rather might be mediated via some non-histone targets. NF-κB has been shown to be involved in the regulation of eosinophil apoptosis [3]. NF-κB assembly with IκB, as well as its DNA binding and transcriptional activity, are regulated by p300/CBP acetyltransferases that principally target Lys218, Lys221 and Lys310 [25-27]. This process is reciprocally regulated by HDACs and several HDAC inhibitors have been shown to activate NF-κB [25-27]. To evaluate whether the effects of HDAC inhibitors could be mediated via acetylation of a non-histone target such as NF-κB, we evaluated the effect of TSA on the acetylation status of NF-κB p65. However, TSA (330 nM) did not enhance acetyl-p65 expression in human eosinophils either in the absence (n = 5; Figure 9) or presence of GM-CSF (n = 2) (data not shown).

### Effect of c-jun-N-terminal kinase and PI3K-Akt pathway inhibitors on TSA-induced apoptosis in human eosinophils

**Figure 10:**
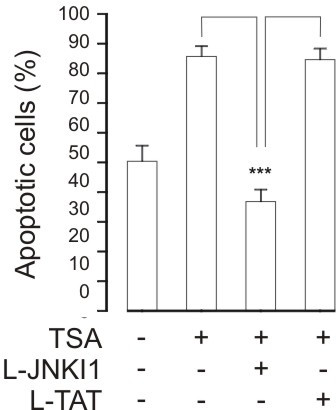
**The effect of the c-jun-N-terminal kinase inhibitor L-JNKI1 (10 μM) on TSA (330 nM)-induced human eosinophil apoptosis**. Apoptosis was assessed by the relative DNA fragmentation assay. Each data point represents the mean ± SEM of 6 independent determinations using eosinophils from different donors. *** indicates p < 0.001 as compared with the respective control (10 μM L-TAT or solvent).

c-jun-N-terminal kinase (JNK) and PI3K-Akt pathways have been proposed to be involved in the modulation of human eosinophil longevity [3,28,29]. To test the involvement of these pathways in HDAC-inhibitor-induced apoptosis, we employd pharmacological inhibitors of JNK and PI3K. Inhibition of JNK activity by the cell permeable inhibitory peptide L-JNKI1 almost completely abolished TSA (330 nM)-enhanced DNA breakdown. In contrast, the negative control peptide L-TAT had no effect (Figure 10).

Inhibition of PI3K-Akt pathway by two chemically distinct inhibitors, namely wortmannin (10-100 nM) and LY294002 (5-50 μM) did not affect TSA-induced apoptosis in human eosinophils (n = 6, data not shown).

### Involvement of caspases in TSA-induced apoptosis in human eosinophils

**Figure 11:**
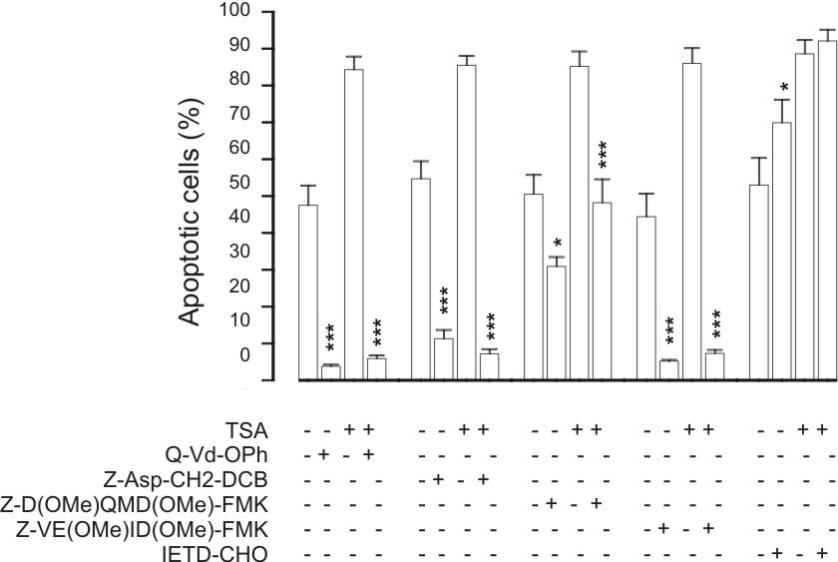
**The effect of caspase inhibitors on TSA (330 nM)-induced human eosinophil apoptosis**. The concentrations used were: Q-Vd-Oph (20 μM), Z-Asp-CH2-DCB and IETD-CHO (100 μM) and Z-D(OMe)QMD(OMe)-FMK and Z-VE(OMe)ID(OMe)-FMK (200 μM). Apoptosis was assessed by the relative DNA fragmentation assay. Each data point represents the mean ± SEM of 6-7 independent determinations using eosinophils from different donors. * indicates p < 0.05 and *** p < 0.001 as compared with the respective control in the absence of caspase inhibitors.

Even though the involvement of caspases in apoptosis in general is well established, surprisingly little is known of the role caspases in human eosinophils [3,30] and the actual caspases mediating apoptosis in human eosinophils remain largely unknown [3,30]. General caspase inhibitors Q-Vd-OPh and Z-Asp-CH2-DCB completely antagonized the effect of TSA on apoptosis in human eosinophils (Figure 11). Inhibitors of caspase 6 (Z-VE(OMe)ID(OMe)-FMK) and 3 (Z-D(OMe)QMD(OMe)-FMK) compeletely and partly antagonized TSA-induced DNA breakdown in human eosinophils, respectively (Figure 11). In contrast, inhibition of caspase 8 (IETD-CHO) had no effect (Figure 11). These results suggest a role for caspases 3 and 6, but not 8, in the mechanism of action of TSA in human eosinophils.

### HDAC inhibitors enhance apoptosis in J774 macrophages

**Figure 12:**
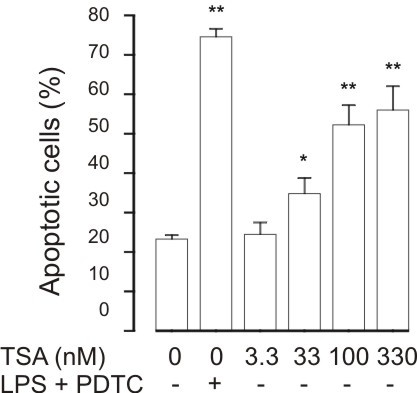
**The effect of HDAC inhibitor Trichostatin A (TSA) on apoptosis in J774.2 macrophages during culture for 24 h**. For comparison, is shown the effect of a known inducer of apoptosis in J774 macrophages, i.e. combination of LPS (10 ng/ml) and PDTC (100 μM). Apoptosis was assessed by flow cytometry measuring the percentage of Annexin V-positive cells. *P < 0.05 and **P < 0.01 as compared with the respective control. Mean ± S.E.M., n = 12.

Macrophages are considered to be important in the removal of apoptotic cells. To evaluate whether HDAC inhibitors could affect macrophage survival, we evaluated the effects of TSA on apoptosis in J774.2 macrophages. TSA increased the percentage of Annexin V-positive cells in J774.2 macrophages in a concentration-dependent manner, although to a lesser extent than a combination of LPS and an inhibitor of NF-κB PDTC (100 μM), previously known to induce apoptosis in macrophages (Figure 12).

## Discussion

In the present study we show that HDAC inhibitors inhibit HDAC acitivity and induce apoptosis in human eosinophils and neutrophils in the absence and presence of survival-prolonging cytokines and glucocorticoids. Furthermore, we report that eosinophils and neutrophils express a different pattern of HDACs, namely the expression of HDAC2 and HDAC9 is higher in neutrophils than in eosinophils and the expression of HDAC8 is higher in eosinophils than in neutrophils. The mechanism of apoptosis-enhancing action of HDAC inhibitors in human eosinophils seems to involve JNK and caspases 3 and 6.

HDAC inhibitors have been reported to cause apoptotic cell death in a variety of cultured transformed cells, including human bladder, breast, prostate, lung, ovary and colon cancers and acute myelogenous leukemia [31]. For example, HDAC inhibitors such as apicidin, sodium butyrate, suberoylanilide hydroxamic acid (SAHA) and TSA have been reported to reduce viability or induce apoptosis in HeLa cells [32,33]. In contrast, normal cells are usually resistant to cell death caused by HDAC inhibitors [9,10] and there is no previous data to describe the effects of HDAC inhibitors on apoptosis in human eosinophils or neutrophils. Supporting our results on the possible anti-inflammatory effects of HDAC inhibitors on granulocytes, recent in vivo data in animals suggest that HDAC inhibitors may have potential to act as anti-inflammatory agents. Choi and coworkers [12] demonstrated that TSA given prophylactically blocked OVA-induced airway hyper-responsiveness, as well as reduced the numbers of eosinophils in lavage fluid [12]. Interestingly, HDAC inhibitors seem not to block the production of eosinophil life-supporting cytokines such as IL-5, but rather may enhance the activity of IL-5 promoter [34]. Thus, it is tempting to speculate that as HDAC inhibitors may not reduce the concentrations of eosinophil survival-prolonging cytokines. The finding that TSA enhances apoptosis in the presence of IL-5- and GM-CSF, may, at least partly, explain the beneficial effects of TSA in models of eosinophilic inflammation.

Structurally distinct HDAC inhibitors were used. Unfortunately, the inhibitory profiles of HDAC inhibitors against all HDAC isoforms have not been thoroughly characterized. TSA has been reported to be a general HDAC inhibitor [31,35-37]. HDAC1 selective inhibitors, MC-1293 [38] and MS-275 at low concentrations [23] did not affect eosinophil apoptosis to a similar extent than TSA or apicidin. This probably excludes HDAC1 as a target of HDAC inhibitors. However, given that the effect of TSA in the HDAC activity assay experiments using nuclear extracts obtained from eosinophils or neutrophils revealed that the HDAC activity was reduced only by 50-60% even at 1 μM suggests either that granulocytes possess a TSA-insensitive HDAC e.g. HDAC4 or 7 or that HDACs are not the major target for HDAC inhibitors in these cells. The EC_50 _values for TSA in enhancing apoptosis in the presence or absence of glucocorticoids were different between eosinophils and neutrophils, whereas no difference was found in the EC_50 _values for TSA in the presence of GM-CSF. This suggests that there may be two or more HDACs responsible mediating these effects or that the effect may reflect the combined effect of two or more HDACs. The expression of HDAC2, HDAC8 and HDAC9 were different between eosinophils and neutrophils. This suggests that one or more of these HDACs may also be involved.

In malignant cell lines activation of caspase cascades as well as changes in the expression of Bcl-2 family members have been described [9,10]. The exact mechanisms how the survival-prolonging cytokines IL-5 and GM-CSF induce eosinophil survival or glucocorticoids induce eosinophil death are not known in detail [3,22,30]. In fact, it is not even known whether glucocorticoid-induced apoptosis involves mainly transcriptional activation or repression [39]. Mechanistically, inhibition of HDAC activity should lead to increased transcription. Treatment with HDAC inhibitors in an in vitro situation leads almost up to 10% of transcriptionally active genes having altered expression [9]. Surprisingly, nearly an equal number of genes are repressed in their expression as those that are activated [9]. Treatment with HDAC inhibitors in vitro causes an increase in the acetylation levels of histones in both normal and tumor cells, including melanocytes and melanoma cell lines [9]. However, normal melanocytes are resistant to cell death caused by HDAC inhibitors, whereas most melanoma cell lines undergo apoptosis [9]. This suggests that the difference between survival and death between normal and malignant cells may be due to acetylation of non-histone proteins rather than histones themselves [9,10]. In eosinophils, NF-κB has been shown to be involved in the regulation of apoptosis [3]. NF-κB assembly with IκB, as well as its DNA binding and transcriptional activity, are regulated by p300/CBP acetyltransferases that principally target Lys218, Lys221 and Lys310 [25-27]. This process is reciprocally regulated by HDACs and several HDAC inhibitors have been shown to activate NF-κB [25-27]. In fact, ineffectiveness of HDAC inhibitors to induce apoptosis in certain cell lines has been proposed to involve the transcriptional activation by acetylation of RelA/p65 subunit of NF-kB through the Akt pathway [26]. However, we were not able to detect any increased acetylation of NF-kB p65 in response to TSA in human eosinophils. Similarly, inhibition of the PI3K-Akt pathway by pharmacological inhibitors did not modulate TSA-induced apoptosis. These results suggest that NF-kB p65 or PI3K-Akt pathway are not involved, but we cannot exclude other non-histone targets.

c-jun-N-terminal kinase (JNK) pathway has been proposed to be involved in spontaneous and nitric oxide- and orazipone-induced apoptosis of human eosinophils [3,16,17,28]. Inhibition of JNK activity by the cell permeable inhibitory peptide L-JNKI1 almost completely abolished TSA-enhanced DNA breakdown, suggesting a role for JNK. Even though the involvement of caspases in apoptosis in general is well established, surprisingly little is known of the role caspases in human eosinophils [3,30] and the actual caspases mediating apoptosis in human eosinophils remain largely unknown [3,30]. General caspase inhibitors Q-Vd-OPh and Z-Asp-CH2-DCB completely antagonized the effect of TSA on apoptosis in human eosinophils similarly to inhibitors of caspases 6 and 3, whereas inhibition of caspase 8 had no effect. However, caspase inhibition also reduced spontaneous apoptosis as previously described [16]. These results suggest a role for JNK and caspases 3 and 6, but not 8, in the mechanism of action of TSA in human eosinophils. This interpretation may be complicated by the fact that the specificity of these inhibitors for caspases 3, 6 and 8 has not been completely characterized. However, neither JNK nor caspases 3 and 6 appear specific for HDAC-inhibitor-induced apoptosis as they have been reported to affect spontaneous or induced apoptosis in human eosinophils [3,16,17,28].

In contrast to the potentiation of glucocorticoid effects in eosinophils, in neutrophils TSA antagonized the survival-prolonging effect of glucocorticoids on neutrophil survival. In addition, the EC_50 _value for TSA for antagonism of glucocorticoid-induced survival in neutrophils was higher than that in eosinophils for enhancement of glucocorticoid-induced apoptosis. One might argue that the effect of HDAC inhibitors is non-specific in that they override the effects of any survival-prolonging factor in granulocytes.

Accumulation, activation and delayed death of neutrophils at the inflamed site has recently been implicated in the pathogenesis of COPD, severe asthma and asthma exacerbations [1]. We found that TSA antagonized GM-CSF-afforded neutrophil survival by inducing apoptosis. In addition, TSA enhanced apoptosis in the absence and presence of glucocorticoids in neutrophils. We were not able to identify any studies exploring the effects of TSA on neutrophilic inflammation in the lung and based on our results such studies are warranted.

HDAC inhibitors are unique in the sense that they antagonize cytokine-afforded survival of eosinophils and neutrophils despite the vast amount of literature that indicates that they are not toxic towards several types of normal non-malignant cell lines [9,10]. In fact, the published phase I-II clinical trials suggest that HDAC inhibitors: 1. inhibit HDAC activity in vivo in humans and 2. show moderate to good tolerability in humans [40-44]. Thus, it is tempting to speculate that HDAC inhibitors might be used to treat also eosinophilic and/or neutrophilic inflammation.

Macrophages are considered to be important in the removal of apoptotic cells. The finding that TSA at similar concentrations induced apoptosis also in a macrophage cell-line suggests that removal of apoptotic cells in the lungs could be impaired. However, in addition to macrophages, lung epithelial cells have been implicated in the removal of apoptotic eosinophils [45] and A549 lung epithelial cells have been reported to be insensitive to apoptosis induced by HDAC inhibitors [26].

## Conclusions

Taken together, our results suggest that HDAC inhibitors such as TSA enhance apoptosis both in the presence and absence of survival-prolonging cytokines in eosinophils and neutrophils. In addition, TSA has an additive effect on apoptosis in the presence of glucocorticoids in eosinophils and antagonizes glucocorticoid-induced neutrophil survival. The mechanism of action in eosinophils involves c-jun-N-terminal kinase and caspases 3 and 6. Thus, HDAC inhibitors have anti-eosinophilic and anti-neutrophilic properties and are possible drug candidates to treat eosinophilic or neutrophilic inflammation.

## List of abbreviations

(COPD): Chronic obstructive pulmonary disease; (GM-CSF): Granulocyte-macrophage colony-stimulating factor; (HAT): Histone acetyltransferase; (HDAC): Histone deacetylase; (IL): Interleukin; (L-JNKI1): GRKKRRQRRR-PP-RPKRPTTLNLFPQVPRSQD-amide; (LPS): Lipopolysaccharide; (L-TAT): RKKRRQRRR-amide, negative control for L-JNKI1; (MC1293): 3-(4-toluoyl-1-methyl-1H-2-pyrrolyl)-N-hydroxy-2-propenamide; (MS-275): [N-(2-aminophenyl)-4-[N-(pyridine-3-ylmethoxy-carbonyl)aminomethyl]benzamide]; (PDTC): ammonium pyrrolidinedithiocarbamate; (TSA): Trichostatin A.

## Competing interests

The authors declare that they have no competing interests.

## Authors' contributions

HK, MJ-J, PI and XZ carried out the eosinophil and neutrophil isolation, flow cytometric assays, morphological analyses, HDAC assays and western blot analysis. UJ gave valuable collaboration in laboratory studies. KI and MI performed the HDAC mRNA assays. KI, IMA and EM participated in the design of the study and helped to draft the manuscript. HK conceived the study, and participated in its design and coordination and drafted the manuscript with XZ. All authors read and approved the final manuscript.
